# Long-term stability of self-reported psychopathic traits in former at-risk adolescents in youth welfare and juvenile justice institutions

**DOI:** 10.1186/s13034-025-00938-w

**Published:** 2025-07-16

**Authors:** H. Hachtel, N. Jenkel, K. Schmeck, M. Graf, J. M. Fegert, M. Schmid, C. Boonmann

**Affiliations:** 1 Department of Forensic Psychiatry, Psychiatric University Hospitals (UPK), Wilhelm Klein-Strasse 27, 4002 Basel, Switzerland; 2 Child and Adolescent Research Department, Psychiatric University Hospitals (UPK) Basel, Basel, Switzerland; 3 Department of Child and Adolescent Psychiatry and Psychotherapy, University Hospital of Ulm, Ulm, Germany; 4 Department of Child and Adolescent Psychiatry, Leiden University Medical Center (LUMC) Curium, Leiden, The Netherlands

**Keywords:** Psychopathic traits, YPI, Adolescents, Long-term stability, Reliable change index, Residential care, Child welfare, Juvenile justice, Internalizing mental health problems, Externalizing mental health problems

## Abstract

**Background:**

The paucity of research examining the long-term self-reported stability of psychopathic traits in adolescents in residential care (both child welfare and juvenile justice system-placed juveniles) and potential influencing factors is a matter of concern. Since psychopathic traits tend to be associated with an earlier onset of delinquent behavior, higher levels of delinquent behavior, and higher rates of recidivism, it is important to study this phenomenon in more detail. The present study aims to examine the long-term stability of psychopathic traits, including its underlying dimensions, in young adults with a history of residential care.

**Method:**

A 10-year follow-up study was conducted, in which a subset of participants who had previously consented to be re-contacted (n = 511) were re-contacted (data collection between 2018 and 2020). A total of n = 203 subjects (average age at follow-up of 25.7, SD = 1.8) completed the questionnaires online, including the Youth Psychopathic Traits Inventory (YPI) (mean time interval between baseline and follow-up; 121 months, SD = 11.7).

**Results:**

At the group level, a significant decrease in psychopathic traits was observed, encompassing the underlying dimensions (i.e., Grandiose-Manipulative [GM], Callous-Unemotional [CU], and Impulsive-Irresponsible [II]).The Reliable Change Index revealed that approximately one-third of adolescents demonstrated a substantial decrease in psychopathic trait symptoms, as well as in GM, CU, and II symptoms. The strongest predictor of psychopathic traits in young adulthood was found to be psychopathic traits at baseline (approximately 10 years earlier).

**Discussion:**

The results of this study suggest a lower long-term stability of self-reported psychopathic traits over a time period of 10 years than was previously assumed in institutionalised youths over a shorter period of time. Research is needed to control for the influence of different aspects of caregiving on outcomes regarding psychopathic traits in order to better interpret these results.

## Introduction

Research over the past two decades has demonstrated the clinical and scientific importance of psychopathic traits (i.e. Grandiose-Manipulative [GM], Callous-Unemotional [CU], and Impulsive-Irresponsible [II] traits [13]) for predicting future delinquent behavior in adolescents [14, 42]. While the stability of psychopathic traits in children and adolescents in residential care has been previously assessed and found to be relatively stable over a 1 year interval [28], there is still scarce evidence of the stability of these traits over longer periods of time. The issue of whether psychopathic traits exhibit high or low stability is of significant clinical importance, as it pertains to the prediction of outcomes, the implementation of preventative measures, and the development of intervention strategies.

Research into the long-term stability of psychopathy-related traits in children and adolescents in residential care found mixed results; a 10 year longitudinal study of individuals formerly in residential care revealed that anger and irritability did not appear to diminish over time [50]. As increased levels of anger and irritability are consistently associated with dimensions of the psychopathic personality concept (i.e. Impulsive-Irresponsible traits) (Fanti et al. [50]) and strongly predict, when stable and present over time, behavioural dysregulation [50], this could point to a greater stability of psychopathic traits. Other studies, however, have noted that the categorical mean-level stability of personality disorders (of which some encompass psychopathic personality traits) can be classified as moderate in young adults with a history of child welfare and juvenile justice placements over a 10 year period [20]. Regarding psychopathic traits, there is evidence that suggests a certain malleability of psychopathic features in the transition from adolescence to adulthood [29].

For better understanding of possible changes or apparent stability at a general level, dimensional observations are important. At the dimensional level, GM traits show greater variability than CU traits among school-aged children [4]. Furthermore, GM traits have been associated with a high level of delinquent development over a 4 year period during adolescence [42]. Regarding CU traits, research has identified CU traits as a potential predictor of negative outcomes over time [9, 41]. A study of over 1200 first-time male adolescent offenders reported that CU traits exhibited a moderate level of stability over a 3 year time course [44]. Consistent with these findings, research across diverse samples has reported that CU traits demonstrate stability similar to other personality traits throughout childhood, adolescence, and early adulthood [3, 38, 52]. In a nutshell, CU traits in youth are often found to be more stable over time compared to other psychopathic traits. This stability is thought to arise from a combination of genetic, developmental, and personality-related mechanisms (i.e. high heritability in boys, shared environmental factors in girls, relational experiences in early childhood, consistent personality features like lack of prosociality that tend to be stable across development) [15, 22, 40], Frick et al. 2008, [53]. In regard to II traits, a Swedish study that assessed 1068 adolescents over a period of four years identified four distinct profiles: three of which were characterized by decreasing levels (n = 804) and another that exhibited moderate stability (n = 264) [47]. At the same time, an investigation of a Swiss sample of 162 adolescents in youth welfare and juvenile justice institutions indicated relative stability of II traits, with approximately 83% of individuals showing no reliable change over a one-year period [28]. In sum, II traits exhibit moderate stability over time, but generally less stability in relation to CU traits. Possible explanations for relative instability of II traits include developmental maturation [6, 47] environmental sensitivity (i.e. II traits are more susceptible to changes in social relationships, parenting quality, and life experiences) Forsmann et al. 2008, [3, 47], and neurobiological differences (i.e. changes in brain networks during adolescence) [11]. To the best of our knowledge, however, no recent studies have examined psychopathic traits at the dimensional level over extended follow-up periods.

In short, evidence describing the longitudinal development of psychopathic personality dimensions over a long period of time is lacking. Therefore, the aim of the current paper is to examine the long-term stability of psychopathic traits, including its underlying dimensions, in young adults with a history in residential care. The present findings extend the observations of the previously published study regarding the stability of self-reported psychopathic traits over time [28]. While the previous mentioned findings suggest a certain temporal stability, the long-term stability is still to be explored. The prediction of possible outcomes in institutional settings from baseline assessments and establishment of preventive measures or treatment modalities will benefit of the longer observation period.

## Methods

### Procedure

The MAZ. study (Model Project for Clarification and Goal-Attainment in Child Welfare and Juvenile Justice Institutions) was conducted between 2007 and 2011 and included 592 children, adolescents, and young adults from 64 different welfare and juvenile justice institutions across the German-, French-, and Italian-speaking regions of Switzerlandchange. Participants were placed in these institutions placed in institutions by court mandate (criminal or protective) or voluntarily (e.g., for educational or vocational support).

Participants, or their legal guardians when required due to age, provided informed consent to participate. Eligibility criteria included sufficient proficiency in German, French, or Italian and an IQ above 70. Data collection took place within the institutions using computerized assessments, including psychometric self- and external evaluations as well as additional questionnaires for socio-pedagogical caseworkers. Ethical approval for the MAZ. study was obtained from the Ethics Committee of Basel (Basel-Stadt and Basel-Landschaft).

Approximately ten years later, the JAEL study (Youth Welfare Trajectories: Learning from Experience, 2018–2020) was conducted to follow up on a subset of the original sample. A total of 511 participants agreed to be recontacted. Despite significant outreach efforts, only 203 of the 511 eligible participants completed follow-up; reasons for attrition are reported in supplemental materials.^1^ JAEL participants also provided informed consent. The study adhered to the Declaration of Helsinki and was approved by the Ethics Commission of Northwestern Switzerland (EKNZ, Ref. 2017–00718).

In both MAZ. and JAEL, face-to-face clinical interviews were conducted. However, as these data are not relevant to the present study, they will not be discussed further.

### Participants

For the present study, to maintain a developmentally homogeneous baseline group and because the YPI was originally designed for adolescents, only participants aged 12–18 at the time of MAZ. (t1) and had completed the Youth Psychopathic Traits Inventory [2] both during MAZ. (t1) and JAEL (t3) were included. The mean time interval between these two assessments was 121.2 months (SD = 11.7). This yielded a final subsample of 143 participants (87 males, 56 females) with a mean age of 25.7 years (SD = 1.8). Table 1 presents the sample characteristics regarding age (at t1), biological sex, Swiss nationality, and type of placement.

**Table 1:** Sample characteristics

	n	%	M	SD
Biological sex				
Male	87	60.8		
Female	56	39.2		
Age				
Male			15.5	1.6
Female			15.7	1.3
Nationality				
Swiss	137	84.6		
Non-Swiss	25	15.4		
Placement type				
Civil law	74	51.7		
Penal law	29	20.3		
Voluntary	40	28.0		

### Measures

#### Psychopathic personality traits in adolescence and young adulthood

Psychopathic personality traits were measured using a computerized version of the Youth Psychopathic Traits Inventory (YPI), a 50-item self-report questionnaire. Each item is rated on a 4-point Likert scale (1 = does not apply at all, to 4 = applies very well). The YPI aligns with a three-dimensional model of psychopathy [12]: the GM dimension, including dishonest charm, manipulation, and grandiosity,the CU dimension, including callousness, unemotionality, and lack of remorse; and the II dimension, including impulsivity, irresponsibility, and thrill-seeking. Higher scores indicate higher levels of these traits. Although the instrument was developed for adolescents (12–18 years old), previous studies have demonstrated its applicability in young adult samples [7, 8]. Our data further support the validity of the YPI for young adults. Internal consistency measured in adolescents (MAZ.) [28] differed only slightly from that found in the present young adult sample (JAEL). For the YPI total score, internal consistency based on Cronbach’s alpha changed from 0.90 to 0.89, for GM from 0.89 to 0.86, for CU from 0.70 to 0.79, and for II from 0.77 to 0.81.

### Mental health problems in adolescence

A computerized version of the Youth Self Report (YSR; [1]) was used in the MAZ. study to measure internalizing and externalizing mental health problems during adolescence. This questionnaire includes approximately 120 behavioral and emotional difficulties common to adolescents. Items are rated on a 3-point Likert scale (0 = not true, 1 = somewhat or sometimes true, 2 = very true or often true). The YSR provides three broadband scales: total problems (TOT), internalizing problems (INT), and externalizing problems (EXT), with scores transformed into T-scores. As described by Hachtel et al. [28], internal consistencies at t1 were good to excellent (α = 0.93 for TOT, α = 0.87 for INT, α = 0.86 for EXT).

### Statistical analyses

In line with our first paper on short-term stability [28], we used the Reliable Change Index (RCI [32, 33],) to assess the stability of psychopathic traits on an individual level. The RCI allows to determine whether observed score changes exceed what would be expected due to measurement error alone. It is typically calculated using the measure’s test–retest reliability and the standard deviation at the first measurement (SD_pre_), providing a standardized metric for individual-level change. However, due to the absence of satisfactory test–retest reliability data for the YPI, we used the scale’s internal consistency (Cronbach’s α) at t1 as a substitute. This approach is acceptable in long-term longitudinal research, where test–retest estimates are often impractical or misleading due to expected developmental and contextual changes over time [24]. Internal consistency thus serves as a conservative and appropriate proxy for estimating measurement error in such contexts [10]. A change is considered statistically significant if it exceeds 1.96 times the Standard Error of the Difference (SE_diff_), which reflects expected variability due to measurement unreliability.

**Fig. 1:**
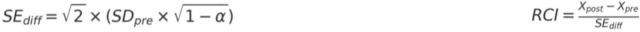
Formula for the standard error of difference (SEdiff, left) and formula for the reliable change index (RCI, right)

The formula for calculating SE_diff_ is shown in Fig. 1 (left). It incorporates the standard deviation of the pre-test scores (SD_pre_) and the internal consistency of the measurement scale (α), such as Cronbach’s alpha.

The corresponding formula for the RCI is shown in Fig. 1 (right). It uses the difference between the post-test score (Xpost) and pre-test score (X_pre_), divided by the SE_diff_ value calculated earlier.

If the absolute value of the RCI exceeds 1.96, the change is considered statistically reliable at the 95% confidence level. Accordingly, reliable change cut-offs were 1.73 for the YPI total score (SD_Pre_ = 2.11, α = 0.90, SE_Diff_ = 0.88), 2.48 for the GM dimension (SD_Pre_ = 2.70, α = 0.89, SE_Diff_ = 1.27), 3.16 for the CU dimension (SD_Pre_ = 2.08, α = 0.70, SE_Diff_ = 1.61), and 3.22 for the II dimension (SD_Pre_ = 2.42, α = 0.77, SE_Diff_ = 1.64). Therefore, all RCI calculations were done per participant per dimension, and any change exceeding the respective threshold in a given dimension was considered a reliable change.

greater than these values in their respective dimensions was considered a reliable change.

Based on these cut-offs, participants were categorized into three groups: a reliable increase (a change larger than the cut-off in the positive direction), a reliable decrease (a change larger than the cut-off in the negative direction), or no reliable change (a change smaller than the cut-off in either direction), for each respective score.

Statistical analyses were performed using SPSS for MAC, version 29. Paired sample t-tests were used to compare YPI means between t1 and t3. Differences between reliable change groups were analyzed using chi-squared tests for categorical variables and ANOVAs for continuous variables, with Bonferroni correction for multiple comparisons. Exploratory multiple linear regression models were also conducted to identify factors influencing YPI total scores and dimensions at t3. Independent variables included YPI scores at t1, age, gender, time between t1 and t3, and adolescent mental health problems at t1. The significance level was set at α = 0.05.

## Results

First, psychopathic trait scores on the YPI were compared between t1 and t3 using means comparisons. The results revealed highly significant differences in the total score as well as in all underlying dimensions (see Table 2).

**Table 2:** Mean differences in YPI mean scores between t1 and t3

	t1 (SD)	t3 (SD)	*p*-value	Cohen’s *d* (CI)
*Total sample (N* = *143)*				
Total mean score	11.35 (2.25)	10.35 (1.70)	<.001	0.46 (0.29, 0.63)
Grandiose-Manipulative	10.39 (2.69)	9.08 (2.16)	<.001	0.45 (0.28, 0.63)
Callous-Unemotional	10.79 (2.61)	9.75 (2.17)	<.001	0.40 (0.23, 0.57)
Impulsive-Irresponsible	12.87 (2.72)	12.22 (2.48)	0.004	0.24 (0.08, 0.41)

**Fig. 2:**
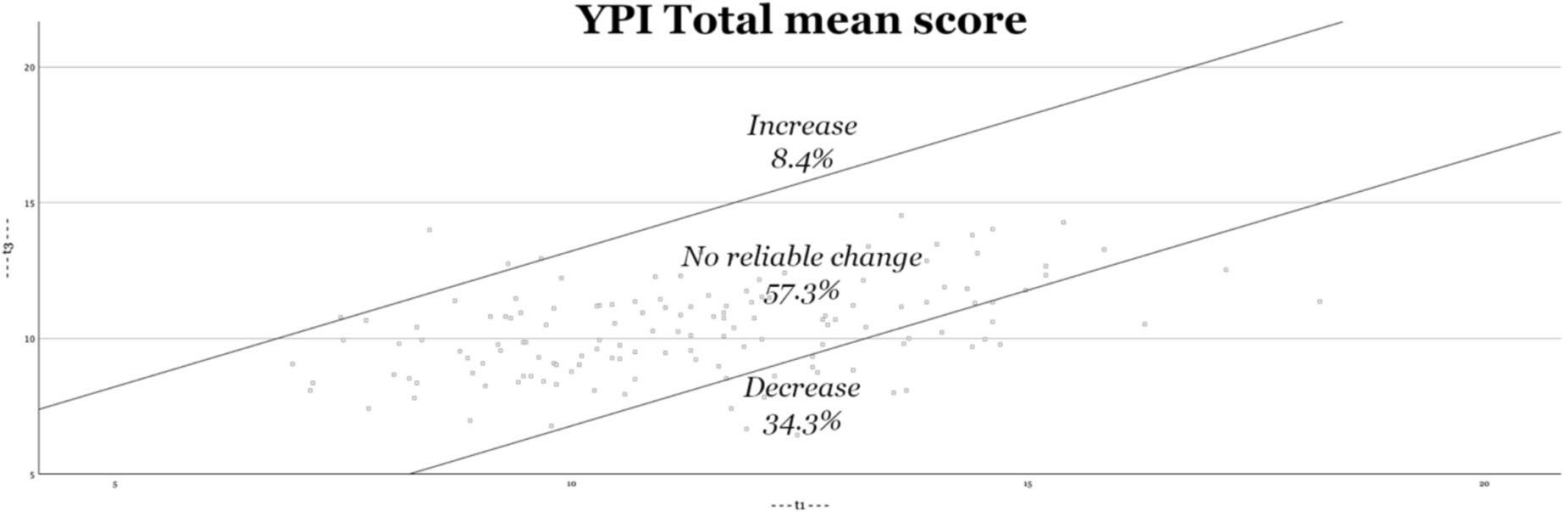
Reliable changes YPI Total score

**Fig. 3:**
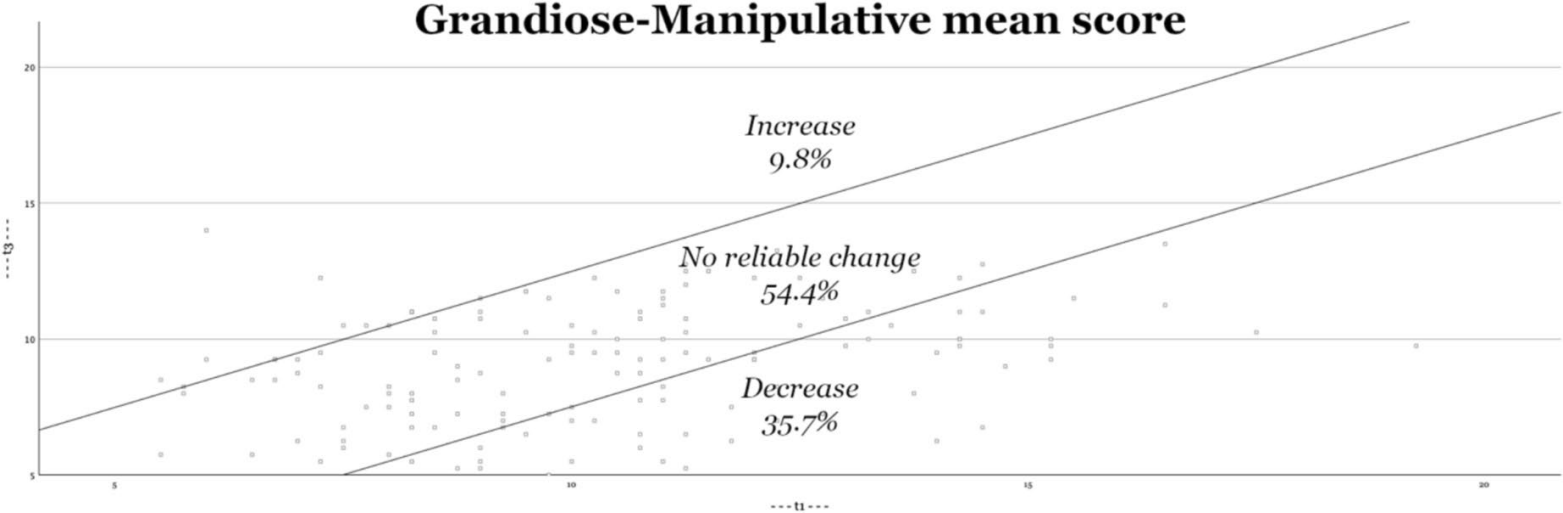
Reliable changes GM traits

**Fig. 4:**
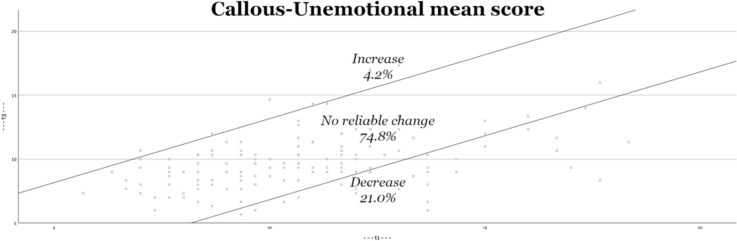
Reliable changes CU traits

**Fig. 5:**
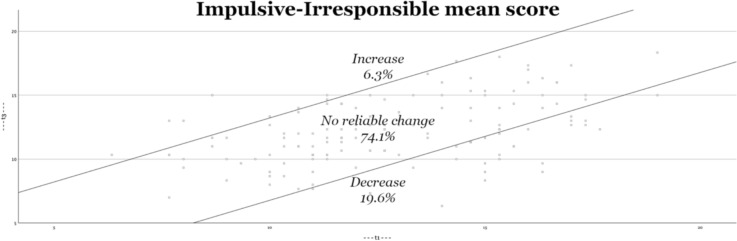
Reliable changes II traits

Second, Figs. 2, 3, 4, 5 visually depict the proportions of reliable change. For both the total score and the interpersonal dimension (GM), approximately one-third of the adolescents show significant improvement (decrease in symptoms). On the CU and II dimensions, approximately one-fifth of adolescents show improvement. The percentage of adolescents who experience deterioration (increase in score) is less than 10% (for both the total score and all underlying dimensions).

Third, Table 3 shows different predictive linear regression models for the total YPI score and its three underlying dimensions at t3. In step 1, only the corresponding outcome at t1 was included (the corresponding YPI score at t1 as a predictor of the corresponding YPI score at t3). In step 2, age (at t1), biological sex, and the time interval between t1 and t3 were added. In step 3, emotional and behavioral problems were added to the model. In all models, the outcome equivalent at t1 was the strongest predictor. Although age for CU and gender for II and total score appears to have a small effect, the R^2^ does not increase significantly with the addition of predictors beyond the YPI baseline score at t1.

**Table 3:** Linear regressions predicting YPI Total mean score and YPI dimensions at t3

		Unstandardized coefficients		Standardized coefficients			
*Step*	*Predictor*	*B*	*SE*	*β*	*p*	*R*^*2*^	*F*
YPI Total mean score at t3							
1						0.462	23.620
	YPI Total mean score at t1	0.355	0.073	0.462	** <0.001**		
2						0.513	7.507
	YPI Total mean score at t1	0.387	0.081	0.504	** <0.001**		
	Age	− 0.202	0.160	− 0.126	0.211		
	Biological sex (1 = male; 2 = female)	− 0.634	0.358	− 0.173	0.080		
	Time span in months (t1-t3)	− 0.016	0.016	− 0.106	0.303		
3						0.542	5.662
	YPI Total mean score at t1	0.273	0.101	0.356	**0.008**		
	Age	− 0.270	0.164	− 0.168	0.103		
	Biological sex (1 = male; 2 = female)	− 0.922	0.410	− 0.251	**0.027**		
	Time span in months (t1− t3)	− 0.016	0.016	− 0.106	0.302		
	YSR INT at t1	− 0.009	0.018	− 0.060	0.613		
	YSR EXT at t1	0.045	0.025	0.272	0.073		
YPI Total Grandiose-Manipulative mean score at t3							
1						0.314	9.545
	YPI Grandiose-Manipulative mean score at t1	0.251	0.081	0.314	**0.003**		
2						0.371	3.353
	YPI Grandiose-Manipulative mean score at t1	0.259	0.088	0.325	**0.004**		
	Age	− 0.142	0.212	− 0.072	0.503		
	Biological sex (1 = male; 2 = female)	− 0.796	0.479	− 0.176	0.100		
	Time span in months (t1− t3)	− 0.017	0.020	− 0.088	0.414		
3						0.404	2.666
	YPI Grandiose− Manipulative mean score at t1	0.205	0.097	0.257	**0.037**		
	Age	− 0.221	0.220	− 0.112	0.319		
	Biological sex (1 = male; 2 = female)	− 0.948	0.543	− 0.210	0.084		
	Time span in months (t1− t3)	− 0.021	0.021	− 0.108	0.318		
	YSR INT at t1	− 0.021	0.023	− 0.114	0.369		
	YSR EXT at t1	0.045	0.029	0.221	0.123		
YPI Total Callous-Unemotional mean score at t3							
1						0.386	15.206
	YPI Callous Unemotional mean score at t1	0.327	0.084	0.386	** < 0.001**		
2						0.464	0.5755
	YPI Callous Unemotional mean score at t1	0.336	0.096	0.396	** < 0.001**		
	Age	− 0.448	0.212	− 0.214	**0.037**		
	Biological sex (1 = male; 2 = female)	− 0.608	0.510	− 0.127	0.236		
	Time span in months (t1− t3)	− 0.011	0.021	− 0.055	0.603		
3						0.466	3.785
	YPI Callous Unemotional mean score at t1	0.320	0.110	0.377	**0.005**		
	Age	− 0.459	0.224	− 0.219	**0.44**		
	Biological sex (1 = male; 2 = female)	− 0.620	0.596	− 0.130	0.301		
	Time span in months (t1− t3)	− 0.011	0.021	− 0.056	0.600		
	YSR INT at t1	− 0.009	0.024	− 0.048	0.700		
	YSR EXT at t1	0.011	0.031	0.050	0.729		
YPI Total Impulsive− Irresponsible mean score at t3							
1						0.563	40.313
	YPI Impulsive− Irresponsible mean score at t1	0.517	0.081	0.563	** < 0.001**		
2						0.588	11.071
	YPI Impulsive− Irresponsible mean score at t1	0.562	0.088	0.612	** < 0.001**		
	Age	2.783E− 5	0.212	0.000	1.000		
	Biological sex (1 = male; 2 = female)	− 0.878	0.489	− 0.167	0.076		
	Time span in months (t1− t3)	− 0.024	0.021	− 0.107	0.267		
3						0.612	8.172
	YPI Impulsive-Irresponsible mean score at t1	0.449	0.113	0.489	** < 0.001**		
	Age	− 0.123	0.220	− 0.053	0.577		
	Biological sex (1 = male; 2 = female)	− 1.221	0.525	− 0.232	**0.022**		
	Time span in months (t1− t3)	− 0.025	0.021	− 0.111	0.245		
	YSR INT at t1	0.013	0.024	0.061	0.589		
	YSR EXT at t1	0.047	0.034	0.201	0.171		

*Note.* SE = standard error of B. Biological sex: 1 = male, 2 = female. Time span (t1− t3) = time interval between t1 and t3 in months. YSR INT = Internalizing problems (*T*-score). YSR EXT = Externalizing problems (*T*-score). Bold values reach the significance level (set at α = 0.05).

Fourth, the three RCI groups (increasers, no reliable change, decreasers) were compared across several relevant variables (Table 4). A significant difference between the groups was in behavioral problems at T1 (YSR EXT). Adolescents who showed reliable improvement (decreases in YPI scores) had higher scores for externalizing problem during the MAZ. study compared to adolescents who showed reliable deterioration (increases in YPI score) or no reliable change (in YPI score). Additionally, regarding CU traits, men were significantly more likely than women to be in the increasers group, while women were more likely to be in the no reliable change group.

**Table 4:** Differences between reliable change groups

	Increasers (%/M, SD)	No reliable change (%/M, SD)	Decreasers (%/M, SD)	Chi^2^/	p
YPI Total mean score					
Biological sex (m, f)	11.5, 3.6	55.2, 60.7	33.3, 35.7	2.787^a^	0.248
Age (at t3)	24.8, 1.6	25.6, 1.9	26.2, 1.7	3.437	0.**035**
Time span in months (t1-t3)	122.6, 8.8	120.2, 11.1	122.6, 13.3	0.768	0.466
Nationality (Swiss, Non-Swiss)	6.7, 8.6	60.0, 57.0	33.3, 34.4	0.084^b^	0.959
Placement type (civil law, penal law, voluntary)	9.5, 3.4, 10.0	54.1, 48.3, 70.0	36.5, 48.3, 20.0	6.885^b^	0.142
YSR INT at t1	52.1, 7.1	60.6, 12.3	60.8, 12.1	1.649	0.198
YSR EXT at t1	540.7, 7.1	61.9, 10.8	67.2, 10.5	4.828	**0.010**
YPI Grandiose-Manipulative mean score					
Biological sex (m, f)	11.5, 7.1	55.2, 53.6	33.3, 39.3	1.013	0.602
Age (at t3)	25.3, 1.8	25.7, 1.9	25.8, 1.8	2.687	0.675
Time span in months (t1-t3)	121.6, 9.4	120.4, 12.1	122.4, 11.8	0.439	0.646
Nationality (Swiss, Non-Swiss)	20.0, 8.6	53.3, 54.7	26.7, 36.7	2.169a	0.338
Placement type (civil law, penal law, voluntary)	10.8, 3.4, 12.5	56.8, 55.2, 50.0	32.4, 41.4, 37.5	2.310^b^	0.679
YSR INT at t1	54.9, 4.9	60.4, 12.3	61.1, 13.2	1.054	0.353
YSR EXT at t1	56.0, 6.2,	62.4, 11.1	66.5, 10.8	3.921	**0.023**
YPI Callous-Unemotional mean score					
Biological sex (m, f)	6.9, 0.0	67.8, 85.7	25.3, 14.3	7.286^b^	**0.026**
Age (at t3)	25.2, 1.9	25.6, 1.9	26.2, 1.8	1.262	0.286
Time span in months (t1-t3)	128.7, 8.4	120.2, 10.8	123.2, 14.4	2.076	0.129
Nationality (Swiss, Non-Swiss)	0.0, 4.7	80.0, 74.2	20.0, 21.1	0.771^b^	0.680
Placement type (civil law, penal law, voluntary)	4.1, 3.4, 5.0	77.0, 62.1, 80.0	18.9, 34.5, 15.0	4.278^c^	0.370
YSR INT at t1	54.8, 5.3	60.6, 12.2	59.1, 12.9	0.503	0.607
YSR EXT at t1	55.0, 9.7	62.3, 10.4	68.0, 11.8	3.263	**0.043**
YPI Impulsive-Irresponsible mean score0					
Biological sex (m, f)	9.2, 1.8	75.9, 71.4	14.9, 26.8	5.503^a^	.064
Age (at t3)	25.8, 1.6	25.6, 1.9	26.1, 1.6	1.028	0.360
Time span in months (t1-t3)	122.4, 10.5	120.7, 11.6	122.9, 12.4	0.450	0.638
Nationality (Swiss, Non-Swiss)	6.7, 6.3	93.3, 71.9	0.0, 21.9	4.119^b^	0.128
Placement type (civil law, penal law, voluntary)	6.8, 6.9, 5.0	68.9, 69.0, 87.5	24.3, 24.1, 7.5	5.628^c^	0.229
YSR INT at t1	52.0, 7.0	60.5, 12.4	61.1, 11.7	1.453	0.239
YSR EXT at t1	58.7, 8.4	61.9, 10.6	69.8, 11.0	4.130	**0.019**

YSR INT = Internalizing problems (T-score). YSR EXT = Externalizing problems (*T*-score). Bold values reach the significance level (set at α = 0.05).

^a^1 cell has an expected frequency less than 5

^b^2 cells have an expected frequency less than 5

^c^3 cells have an expected frequency less than 5

## Discussion

The present study examined changes in self-reported psychopathic traits, including its underlying dimensions, in at-risk youths in institutional settings over an observation period of at least 10 years. Previous findings over a shorter period of approximately 11 months indicated relatively greater stability in total score and underlying dimensions of psychopathic traits in youth [28]. In contrast, over the long term, both the YPI total score and the three underlying dimensions differed significantly. This was also reflected in the RCI, where a larger group reported improvement at 10 years than at 11 months. These results suggest that in high-risk institutionalized populations, psychopathic traits may show greater malleability over longer time spans than previously assumed, both at the global level as well as the dimensional level. This is partly in contrast to the observations of psychopathic traits in a North American sample over substantial time periods in children and adolescents, which described a high stability [38], and a 6 year follow-up study in a Spanish sample of children aged 6–11 at onset, which described moderate to high stability of overall psychopathic traits [37]. The differences of results in the mentioned studies may partly be explained by the different types of informants (self-reports vs. third-party-reports), as well as the different samples (institutionalized youth vs. general school samples). Of note, in our results, the time span between t1 and t3 had non-significant effects on outcome scores in the linear regression models suggesting that the amount of time alone may not fully explain the changes observed. Therefore, treatment and specific institutional influences may account for the greater reduction in psychopathic traits in the present sample. Furthermore, aspects of re-parenting of traumatized adolescents and the possibility of corrective relationship experience in institutions might have protective effects in the long-run [5, 17]. This is consistent with findings indicating that problem severity at baseline is predictive of improvement in externalizing problems over the course of treatment [27]. The current results imply that baseline behavioral and interpersonal severity predicts greater trait change and would underline the assumption, that burdened adolescents displaying behavioral problems [GM and II] and high CU traits benefit most from institutional interventions in regard to psychopathic traits. Although we lack direct data on institutional interventions, the decline in traits, particularly among those with higher baseline behavioral severity, raises the possibility that structured environments and corrective relational experiences may have played a protective role. In addition, the current sample was significantly older than the aforementioned sample at t1, which may represent a general ameliorative effect of age on problematic interpersonal, affective and behavioral dimensions [26]. While other research has suggested considerable stability of psychopathic traits in adolescents, including GM traits [36], our results suggest greater malleability of GM traits than CU and II traits. This may underscore the importance of maturation with respect to aspects of narcissism and Machiavellianism as expressed in GM traits [54]. II traits in youth are moderately stable but show more change over time than CU traits, largely due to greater environmental influence and developmental progression (Bloningen et al. 2006, [3]). Callous-unemotional (CU) traits tend to persist more over developmental periods than Grandiose-Manipulative (GM) or II traits as affective insensitivity seems to be neurologically rooted and as such less shaped by external factors [31, 53]. Furthermore, we may be representing transient developmental parallels to psychopathic traits, i.e., “transient features of a developmental process that will not be, as he or she reaches adult maturity.” [49], p.224).

### Limitations

Measurement-related limitations include the modality of assessment, namely self-reports on psychopathic traits. While self-reports offer the advantage of being more ecological and potentially more insightful, external assessments by parents and teachers are more objective. Further, CU may be particularly susceptible to underreporting due to limited emotional insight or impression management. Furthermore, the behavioral scale in particular is very closely associated with many other mental illnesses. The self-reports will probably favour a longitudinal regression to the middle more strongly than blinded judgments of others (Barnett et al. 2005).

Sample-related limitations are the high attrition rate, and Swiss-specific system limiting generalizability. Notably, Switzerland permits the management of juveniles under both criminal and civil law decisions within the same institutions, which may result in interactions (for further details, see [34]. Consequently, the present Swiss results may not be transferable to other countries and legal systems. Consequently, further research in diverse international contexts is imperative to enhance our understanding of these phenomena. Also, the heterogeneity of the sample, which necessitates subgroup analyses. However, the size of the sample often does not allow for this. More in-depth analysis of subgroups was prevented by small cell sizes and therefore no more specific inferences could be made. The low number of participants could be the possible consequence of the very characteristics of the sample (i.e., not easily accessible target group of adolescents at risk) and the study design (extensive assessment over repeating measurement time points) in the environment of residential youth care (for an overview of the study design, see [48]).

Design-related limitations include the missing assessment of substance misuse in the present study. There is a consistent association between substance misuse and psychopathic traits, as well as the persistence of antisocial behavior [16, 51]. Therefore, the absence of an assessment may introduce a selection bias. Additionally, the long-term observational periods in the present study encompass a multitude of influences and events that are not recorded on an individual level, thereby precluding specific inferences. The lack of treatment data is a further restriction for the interpretation of reliable changes.

### Implications

The long-term outcome of less stability of psychopathic traits in youth may allay concerns about the negative adult outcomes, i.e., chronic offending trajectories, impaired levels of psychosocial functioning, and self-harming behaviors (e.g., substance use) [16, 30, 35]. This may foster an attitude of *doing nothing*, especially regarding psychopathic traits, and letting maturation run its course [39]. From a clinical perspective, it is well known that some therapeutic interventions, especially for personality disorders, take time to take effect [43]. Thus, the short-term stability of psychopathic traits is not surprising. The influence of institutions and professional caregivers would take time, and the approach of doing nothing would be detrimental to the general goal of preventing further harm to others and the adolescent in question [46]. Another hypothesis could be that psychopathic traits are a resource, i.e., an adaptive function in a hostile environment or adverse situation. This would imply a protective function of psychopathic personality traits [45]. This concept would therefore characterize psychopathic traits as a potential beneficial factor for overcoming harmful or detrimental periods with the possibility of reducing the severity of psychopathic traits according to circumstances and social environment. Therefore, psychopathic traits could be understood as a survival strategy to adapt to one's environment, but which also has side effects and developmental risks. As these side effects have clear potential for severe harm of one-self and others, care must be taken not to silently endorse this disruptive developmental path. This is especially relevant in institutional care, where the goal is both rehabilitation and protection of others.

## Conclusions

Our findings suggest less long-term stability of self-reported psychopathic traits over a 10-year period than previously suggested for institutionalized adolescents [28]. One possible implication for clinical practice would be the recurrent assessment of psychopathic traits over the developmental course of individuals identified as at-risk in adolescence. Therefore, we recommend annual YPI assessments during institutional stays, and gathering of information from different sources to add external viewpoints to strengthen the validity of assessments (e.g. using the Psychopathy Checklist– Youth version). More research is needed that controls for the stability of psychopathic traits in other samples (e.g., school samples) in terms of total scores and dimensions over time. Future research should be initiated to control for the influence of different aspects of caregiving on outcomes related to psychopathic traits (e.g., therapeutic settings vs. parental care, etc.). As well, emphasis should be placed on developing and better understanding the interplay between evidence-based psychotherapeutic interventions and milieu therapeutic interventions specifically with regard to psychopathic personality traits, and coming to know what works and why.

## Data Availability

The datasets generated and/or analysed during the current study are not publicly available due [property of the federal ministry of justice] but are available from the corresponding author on reasonable request.

## Footnotes

1 The recruitment of study participants proved to be a challenge. The mean number of attempts to establish contact was 17 per person, with methods including phone calls, letters, packages, emails, and text messages. Participants were offered compensation of up to CHF 500 in retail vouchers. However, eight individuals could not be traced, and eight had died. A further 121 subjects did not respond to our attempts to establish contact. Of the 374 individuals who were contacted, 99 declined for various reasons, including emotional distance, living abroad, or the effort involved. Of the participants who initially consented to partake in the study, 44 did not commence the online assessment, while 28 did not complete it. This resulted in a total of 203 participants successfully completing the online assessment battery. The JAEL sample did not differ from other MAZ. participants in terms of psychosocial demographic characteristics (d'Huart, D., Hutsebaut, J., Seker, S., Schmid, M., Schmeck, K., Burgin, D., & Boonmann, C. (2022). Personality functioning and the pathogenic effect of childhood maltreatment in a high-risk sample. *Child Adolesc Psychiatry Ment Health*, *16*(1), 95. https://doi.org/10.1186/s13034-022-00527-1; d'Huart, D., Steppan, M., Seker, S., Burgin, D., Boonmann, C., Birkholzer, M., Jenkel, N., Fegert, J. M., Schmid, M., & Schmeck, K. (2022). Prevalence and 10-Year Stability of Personality Disorders From Adolescence to Young) Adulthood in a High-Risk Sample [Original Research]. *Front Psychiatry*, *13*, 840678. https://doi.org/10.3389/fpsyt.2022.840678
